# Long‐term impact of COVID‐19 infection on cognitive dysfunction among Chinese older adults: a national cross‐sectional online study

**DOI:** 10.1002/alz.088880

**Published:** 2025-01-09

**Authors:** Qing Huang, Jingnan Wu, Liming Li, Shi Yao, Yan Wang, Yatian Li, Lili Cui

**Affiliations:** ^1^ Guangdong Key Laboratory of Age‐Related Cardiac and Cerebral Diseases, Affiliated Hospital of Guangdong Medical University, Zhanjiang, Guangdong China; ^2^ Shanghai Bestcovered Limited, Shanghai China; ^3^ The Marine Biomedical Research Institute of Guangdong, School of Ocean and Tropical Medicine, Guangdong Medical University, Zhanjiang, Guangdong China

## Abstract

**Background:**

The effects of the SARS‐CoV‐2 virus (COVID‐19) on the central nervous system predispose to cognitive dysfunction in older adults. However, limited information is available on how long this impact will last. This study aims to assess the cognitive decline in Chinese older adults after COVID‐19 using a cross‐sectional study.

**Method:**

A nationwide cross‐sectional study was conducted among 1698 individuals over 50 years old who completed an online questionnaire survey on their cognitive impairment. Based on the Subjective Cognitive Decline Questionnaire (SCD‐Q), we designed a self‐administration SCD‐18 questionnaire that assess perceived subjective decline in attention, comprehension, memory, emotion, motivation and sleep. The scores of each evaluation index in the SCD‐18 questionnaire ranges from 0 to 6, with higher scores indicating a greater possibility of cognitive dysfunction. We compared differences in relevant cognitive function between different participants and performed a multiple linear regression analysis to identify factors that might be associated with cognitive function.

**Result:**

Older adults who were infected with COVID‐19 had significantly higher SCD‐18 scores than uninfected participants (*P* = 0.005). Among the infected participants, the SCD‐18 scores of females were significantly higher than that of males (*P* = 0.007), especially in memory (*P*<0.001), and rural participants had higher SCD‐18 scores than urban participants (*P*<0.001). Specifically, the SCD‐18 scores of the infected participants for 1‐6 months were significantly higher than that of the uninfected participants (*P* = 0.003), especially in comprehension (*P* = 0.011), memory (*P*<0.001), motivation (*P* = 0.016), and sleep (*P* = 0.008). Participants infected for more than 6 months did not have significantly different SCD‐18 scores from uninfected participants (*P* = 0.118), suggesting that cognitive function gradually recovered. Regression analysis indicated that female and rural area were associated with cognitive impairment.

**Conclusion:**

The cognitive function of older adults due to the COVID‐19 pandemic has changed over time. Female and rural area were the risk factors for cognitive decline. Therefore, further long‐term interventions for vulnerable older adults with a history of COVID‐19 should be considered to maintain cognitive health.

